# Novel, Divergent Simian Hemorrhagic Fever Viruses in a Wild Ugandan Red Colobus Monkey Discovered Using Direct Pyrosequencing

**DOI:** 10.1371/journal.pone.0019056

**Published:** 2011-04-22

**Authors:** Michael Lauck, David Hyeroba, Alex Tumukunde, Geoffrey Weny, Simon M. Lank, Colin A. Chapman, David H. O'Connor, Thomas C. Friedrich, Tony L. Goldberg

**Affiliations:** 1 Department of Pathology and Laboratory Medicine, University of Wisconsin-Madison, Madison, Wisconsin, United States of America; 2 Jane Goodall Institute, Entebbe, Uganda; 3 Kibale EcoHealth Project, Makerere University Biological Field Station, Kanyawara, Uganda; 4 Wisconsin National Primate Research Center, Madison, Wisconsin, United States of America; 5 Department of Anthropology and School of Environment, McGill University, Montreal, Quebec, Canada; 6 Department of Biology, Makerere University, Kampala, Uganda; 7 Department of Pathobiological Sciences, University of Wisconsin-Madison, Madison, Wisconsin, United States of America; Global Viral Forecasting Initiative, United States of America

## Abstract

**Background:**

Simian hemorrhagic fever virus (SHFV) has caused lethal outbreaks of hemorrhagic disease in captive primates, but its distribution in wild primates has remained obscure. Here, we describe the discovery and genetic characterization by direct pyrosequencing of two novel, divergent SHFV variants co-infecting a single male red colobus monkey from Kibale National Park, Uganda.

**Methodology/Principal Findings:**

The viruses were detected directly from blood plasma using pyrosequencing, without prior virus isolation and with minimal PCR amplification. The two new SHFV variants, SHFV-krc1 and SHFV-krc2 are highly divergent from each other (51.9% nucleotide sequence identity) and from the SHFV type strain LVR 42-0/M6941 (52.0% and 51.8% nucleotide sequence identity, respectively) and demonstrate greater phylogenetic diversity within SHFV than has been documented within any other arterivirus. Both new variants nevertheless have the same 3′ genomic architecture as the type strain, containing three open reading frames not present in the other arteriviruses.

**Conclusions/Significance:**

These results represent the first documentation of SHFV in a wild primate and confirm the unusual 3′ genetic architecture of SHFV relative to the other arteriviruses. They also demonstrate a degree of evolutionary divergence within SHFV that is roughly equivalent to the degree of divergence between other arterivirus species. The presence of two such highly divergent SHFV variants co-infecting a single individual represents a degree of within-host viral diversity that exceeds what has previously been reported for any arterivirus. These results expand our knowledge of the natural history and diversity of the arteriviruses and underscore the importance of wild primates as reservoirs for novel pathogens.

## Introduction

Simian hemorrhagic fever virus (SHFV) was discovered during an “explosive” outbreak of hemorrhagic disease in rhesus macaques (*Macaca mulatta*) in 1964 [1]. Since then, sporadic epidemics have occurred in macaques, likely as a result of contact with, or iatrogenic transmission from, infected but asymptomatic captive African monkeys [2], [3], [4]. Viruses related to SHFV have not yet been found to infect humans, but the clinical manifestations of SHFV resemble those of other primate-associated hemorrhagic fever viruses significant for human health (e.g. Ebola, Marburg, Yellow fever), making SHFV of interest with respect to emerging infectious disease surveillance and biodefense [4], [5]. Although asymptomatic SHFV infections in captive patas monkeys (*Erythrocebus patas*), vervet monkeys (*Cercopithecus aethiops*), and baboons (*Papio* sp.) suggest that such species could be natural SHFV reservoirs [2], [3], the virus has never, to our knowledge, been found in a primate in the wild.

SHFV is in the family *Arteriviridae*, which also contains equine arteritis virus (EAV), lactate dehydrogenase elevating virus of mice (LDV), and porcine reproductive and respiratory syndrome virus (PRRSV) [6]. Similar to the other arteriviruses, SHFV has a positive-sense RNA genome approximately 15 kb in length, consisting of a short 5′ untranslated region, two large open reading frames (ORFs 1a and 1b) encoding the replicase plus various non-structural proteins, a number of smaller downstream ORFs encoding structural proteins, and a short 3′ untranslated region [7]. However, SHFV is unusual compared to the other arteriviruses in that it contains three additional ORFs (2a, 2b, and 3) immediately downstream of the replicase-encoding ORFs, perhaps as the result of a gene duplication event [8]. As of the time of this writing, only one full SHFV genome was available in GenBank (accession number NC_003092), representing prototype strain LVR 42-0/M6941, which was isolated on MA-104 cells from a moribund rhesus macaque during the original SHFV outbreak in 1964 [9]. Because the genome organization of SHFV was determined from this single strain, and because this sequence was generated from an isolate obtained from a macaque after growth in cells derived from embryonic rhesus macaque kidney tissue, it is unclear whether the unusual 3′ genomic architecture of SHFV is typical or whether it might be an artifact of passage in a non-natural host or in cultured cells.

Here we report the discovery of two novel and highly divergent SHFV variants co-infecting a single male red colobus monkey (*Procolobus rufomitratus tephrosceles*) from Kibale National Park, Uganda. Our finding of novel, divergent SHFV variants dually infecting a wild primate sheds new light on the natural history and diversity of SHFV. In addition, using direct pyrosequencing, we were able to recover nearly complete viral genome sequences with only minimal PCR amplification. Our results therefore also confirm the authenticity of SHFV's unique genomic architecture during natural infection.

## Materials and Methods

### Ethics Statement

All animal use followed the guidelines of the Weatherall Report on the use of non-human primates in research and was approved by the Uganda Wildlife Authority, the Uganda National Council for Science and Technology, and the University of Wisconsin Animal Care and Use Committee prior to initiation of the study. Biological materials were shipped internationally under CITES permit #002290 (Uganda).

### Study site and sample collection

Kibale National Park, Uganda, is a semi-deciduous, mid-altitude forest of 795 km^2^ located in western Uganda near the foothills of the Rwenzori Mountains and is notable for having one of the world's highest species diversities (13 primate species) and densities of primates, including the world's densest population of red colobus [10], [11]. On February 5, 2010, a wild adult male red colobus in Kibale was captured as part of a larger study of primate ecology, health, and conservation. The animal was anesthetized with a combination of Ketamine (5.6 mg/kg) and Xylazine (1.7 mg/kg) administered intramuscularly using a variable-pressure air rifle (Pneudart, Inc, Williamsport, PA, USA). Blood was drawn from the femoral vein into an evacuated plasma collection tube (Becton, Dickinson and Company, Inc, Franklin Lakes, NJ, USA) and kept cool until processing. The animal was given a reversal agent (Atipamezole, 0.6 mg/kg), and released after recovery back to its social group without incident. Blood was separated using centrifugation in a field laboratory and frozen immediately in liquid nitrogen for storage and transport to the United States. Samples were shipped in an IATA-approved dry shipper to the USA for further analysis at the Wisconsin National Primate Research Center.

### Molecular methods

One ml of plasma was centrifuged at 5,000×g at 4°C for 5 min with subsequent filtration of the supernatant through a 0.45-µm filter (Millipore, Billerica, MA, USA) to remove residual host cells. Viral RNA was isolated using the Qiagen QIAamp MinElute virus spin kit (Qiagen, Hilden, Germany) according to the manufacturer's instructions, except that carrier RNA was omitted. The eluted RNA was treated with DNase I (DNA-free, Ambion, Austin, TX, USA), and double stranded DNA was generated using the Superscript double-stranded cDNA Synthesis kit (Invitrogen, Carlsbad, CA, USA) primed with random hexamers. DNA was purified using the Agencourt Ampure XP system (Beckman Coulter, Brea, CA, USA) and approximately 1 ng of DNA was subjected to simultaneous fragmentation and adaptor ligation (“tagmentation”) with the Nextera DNA Sample Prep Kit (Roche Titanium-compatible, Epicentre Biotechnologies, Madison, WI, USA). DNA was subsequently cleaned using the Agencourt Ampure XP system, briefly PCR amplified with primers to add Roche/454 titanium-based adaptors onto each fragment (15 cycles), and cleaned again with the Agencourt Ampure XP system. DNA fragments were then sequenced using the GS Junior pyrosequencing system (Roche 454 Life Sciences, Branford, CT, USA).

To analyze pyrosequencing data, raw sequences were first screened to remove redundancy using Galaxy software (http://usegalaxy.org). The remaining reads were then imported into CLC Genomics Workbench (CLC bio, Aarhus, Denmark), trimmed to remove Nextera-specific transposon sequences as well as short and low quality reads, and assembled using the CLC *de novo* assembler. Both singleton and assembled contiguous sequences (contigs) were queried against the GenBank database (http://www.ncbi.nlm.nih.gov/GenBank) using the basic local alignment search tools blastn and blastx [12], with an e-value cut-off of 10 and word sizes of 11 and 3 for blastn and blastx queries, respectively.

Once initial non-host contigs were assembled, a short sequence gap between two aligned fragments was filled by conventional PCR and Sanger sequencing. Briefly, PCR amplicons were generated with the SuperScript III One-Step RT-PCR System (Invitrogen, Carlsbad, CA, USA) according to the manufacturer's protocol, with specific primers designed based on pyrosequence data. Thermocycling conditions included cDNA synthesis at 50°C for 30 min and denaturation at 95°C for 2 min, followed by 40 cycles of denaturation at 94°C for 2 min, annealing at 55°C for 30 s, and extension at 68°C for 1 min. A similar sequence gap at the 3′ terminal open reading frame of one contig was filled by 3′ RACE according to the manufacturer's protocol (Invitrogen, Carlsbad, CA, USA). Briefly, first-strand cDNA synthesis with an oligo(dT)-containing adaptor primer was performed at 42°C for 50 min, followed by amplification of the target cDNA with Platinum Taq DNA polymerase (Invitrogen, Carlsbad, CA, USA) using a specific primer designed based on pyrosequencing data as well as the abridged universal amplification primer (AUAP) (5′ GGCCAGGCGTCGACTAGTAC). Thermocycling conditions included an enzyme activation step of 3 min at 94°C followed by 35 cycles of denaturation at 94°C for 30 s, annealing at 55°C for 30 s, and extension at 72°C for 1 min. Specific primer sequences are available upon request. Amplification products were run on 1% agarose gels, purified (MinElute, Qiagen, Hilden, Germany), and directly sequenced with the DYEnamic ET Terminator Cycle Sequencing Kit (GE Healthcare, Little Chalfont, United Kingdom) on an ABI 3730 Genetic Analyzer (Perkin-Elmer Applied Biosystems, Foster City, CA, USA).

### Phylogenetic analyses

To estimate the similarity of newly discovered SHFV variants to the type strain and to prototypical arteriviruses, nucleic and amino acid sequences of individual ORFs were aligned using the MAFFT method [13], alignments were concatenated, and uncorrected percent sequence identities for the resulting full-length coding genomes were calculated using the computer program MEGA4 [14]. To determine the phylogenetic relationships of newly discovered SHFV variants to known arteriviruses, nucleotide sequences of homologous ORFs in SHFV, EAV, LDV, and PRRSV (1a, 1b, 2a, 2b, 3, 4, 5, 6, and 7, with reference to the EAV genome) were compiled from the literature, with strains chosen from available full-length genome sequences to represent the known within-species diversity of each virus. Non-overlapping coding regions were aligned using a codon-guided version of the MAFFT method [13] with poorly aligned sites removed using the Gblocks alignment cleaning method [15] implemented in the computer program TranslatorX [16]. Phylogenetic analyses were then conducted on concatenated, non-overlapping regions of aligned, cleaned ORFs, using first and second codon positions only, to avoid error associated with third-codon position saturation. Phylogenetic trees were constructed using the maximum likelihood method implemented in the computer program PAUP* 4.0 [17]. The substitution model used in the analysis was estimated using jModelTest [18] and was of the form GTR+I+Γ with the following parameters: nucleotide frequencies of A = 0.2386; C = 0.2413; G = 0.2633; T = 0.2568; substitution rates of AC = 2.5732, AG = 2.7382, AT = 1.4815, CG = 1.6751, CT = 2.1065, and GT = 1; I (proportion of invariant sites) = 0.1330; and Γ (gamma distribution of among-site rate variation) = 2.144, with 4 rate categories. Robustness of phylogenetic groupings was assessed in PAUP* using 1000 bootstrap replicates of the data [19]. New SHFV sequences were deposited in GenBank under accession numbers HQ845737 (SHFV-krc1) and HQ845738 (SHFV-krc2).

## Results

Direct pyrosequencing of RNA extracted from a blood plasma sample of a wild adult male red colobus from Kibale National Park, Uganda, generated 77,891 reads of 280 bp average length, consisting of 19,285 host reads (24.8%) and 58,606 non-host reads (75.2%). *De novo* assembly of singleton non-host reads resulted in a 15.5 kb contiguous sequence (contig) that aligned to the SHFV type strain LVR 42-0/M6941 (GenBank accession number NC_003092.1). A reference assembly against the 15.5 kb contig yielded 35,120 sequence reads with full coverage across the entire contig. Two additional contigs with lengths of 13 kb and 1.9 kb also aligned to the SHFV genome but were highly divergent from the initial 15.5 kb contig. The positions of these additional aligned contigs relative to the SHFV genome suggested that the two fragments might belong to the same viral variant. Using one step RT-PCR and 3′ RACE, respectively, we were able to fill the gap between these two contigs and extend the 3′ end of the contig to complete the terminal open reading frame (ORF9), yielding a new complete contig of 15.2 kb. A reference assembly against this 15.2 kb contig yielded 1,068 sequence reads with full coverage across the entire contig. Initial alignments indicated that both contigs covered all ORFs of the SHFV type strain and extended partially into the 3′ and 5′ untranslated regions. The 15.5 kb and the 15.2 kb contigs were designated SHFV-krc1 and SHFV-krc2, respectively, to indicate their origins in Kibale red colobus and to reflect nomenclature previously used to describe novel simian retroviruses in this same population of red colobus [20].

**Figure 1:**
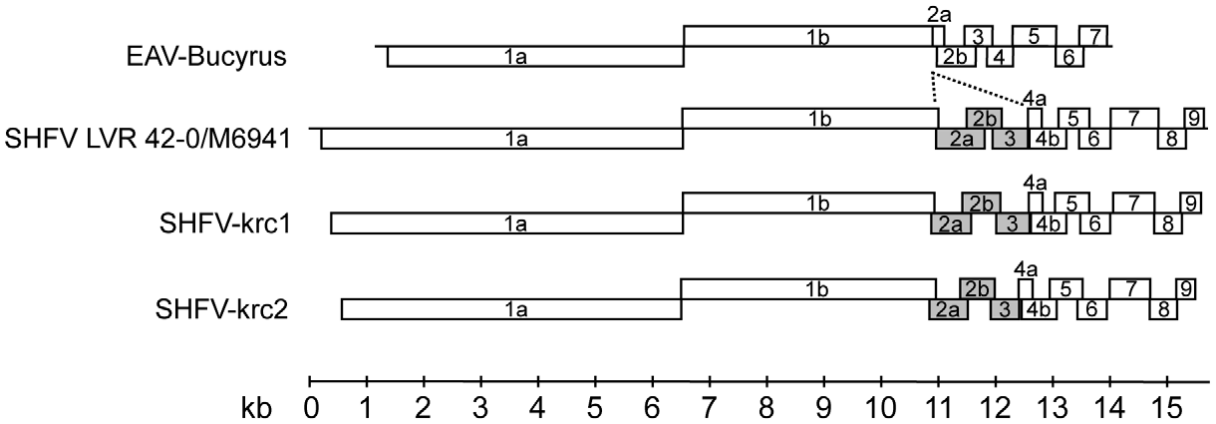
Genome organization of novel simian hemorrhagic fever viruses from a Ugandan red colobus monkey. The novel variants SHFV-krc1 and SHFV-krc2 are shown in comparison to the SHFV type strain LVR 42-0/M6941 and the prototype *Arterivirus*, equine arteritis virus (EAV-Bucyrus strain). Boxes represent open reading frames and are drawn to scale. Shaded boxes indicate ORFs unique to SHFV, with dashed lines indicating the location of putative insertion relative to the EAV genome.

Because we generated near full-length viral genome sequences, we were able to reconstruct the genomic architecture of SHFV-krc1 and SHFV-krc2 (Figure 1). SHFV-krc1 and SHFV-krc2 both possess two large putative replicase-encoding ORFs and several smaller downstream ORFs, all comparable in size to the published ORFs of type strain LVR 42-0/M6941 but all highly divergent at the amino acid level from the type strain (Table 1). Consistent with previously described arteriviruses, the putative ORF1a and ORF1b coding regions in both new SHFV variants contain a canonical heptanucleotide “slippery sequence” (UUUAAAC) and predicted downstream pseudoknot structure [7]. Importantly, both new viral genomes contain homologs of the type strain SHFV ORFs 2a, 2b, and 3 in the same genomic positions, indicating conservation of the unusual 3′ genomic architecture of SHFV even among highly divergent variants.

**Table 1:** Comparison of SHFV open reading frames between the type strain (LVR) and two new variants from Kibale red colobus (krc1 and krc2).

	Length (amino acids)			Percent identity (amino acids)^a^		
ORF^b^	LVR	krc1	krc2	LVR - krc1	LVR - krc2	krc1 - krc2
1a	2105	2048	1978	39.7	40.6	40.1
1b	1491	1464	1485	57.8	58.1	59.3
2a	281	232	223	28.6	34.2	32.3
2b	204	220	202	37.3	30.5	38
3	205	194	166	29.5	32.5	35.2
4a	80	80	81	51.2	53.7	52.5
4b	214	205	204	28.6	34.2	32.3
5	179	199	192	15.6	27.5	25.9
6	182	173	171	31.4	23.1	29.6
7	278	240	236	61.0	56.8	52.6
8	162	161	160	62.7	65.0	69.4
9	111	119	110	45.9	36.5	44.5

a Values are uncorrected percent amino acid identities.

b ORF numbers refer to the SHFV type strain LVR 42-0/M6941 as shown in Figure 1.

**Figure 2:**
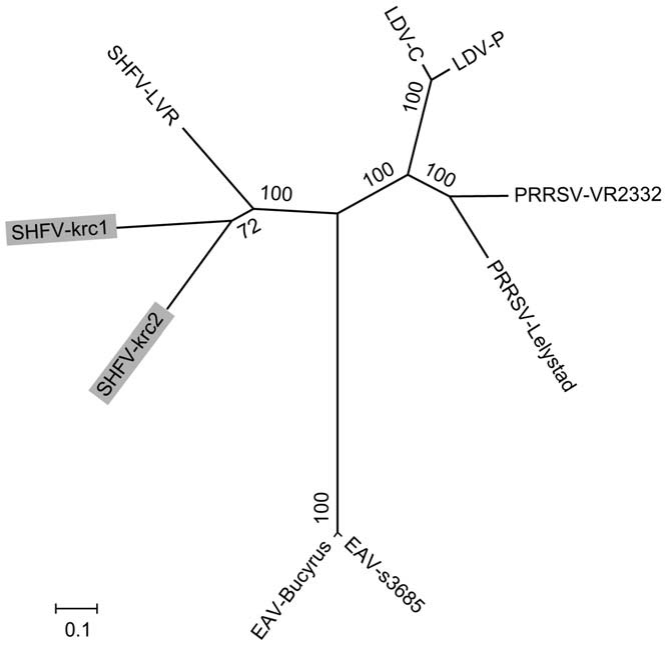
Phylogenetic tree of newly discovered simian hemorrhagic fever viruses and other arteriviruses. The novel variants SHFV-krc1 and SHFV-krc2 are highlighted. Other viruses included in the analysis were chosen to represent the diversity within each viral species based on available full-genome sequences (GenBank accession numbers in parentheses): SHFV-LVR, the SHFV type strain LVR 42-0/M6941 (NC_003092.1); PRRSV-Lelystad, the European (type 1) type strain (M96262.2); PRRSV-VR2332, the North American (type 2) type strain (U87392.3); EAV-Bucyrus strain (NC_002532.2); EAV-s3685 strain (GQ903794.1); LDV-P, the Plagemann strain (U15146.1); and LDV-C, the neuro-virulent strain (L13298.1). This unrooted tree was the likeliest of all trees (−ln L 41,541) found during a maximum likelihood branch-and-bound search with the computer program PAUP* 4.0 [17]. Numbers beside internal nodes indicate statistical support for individual clades (percent), based on 1000 bootstrap replicates of the data. The scale bar indicates genetic distance (nucleotide substitutions per site).

Across the coding genome, the SHFV-krc1 and SHFV-krc2 showed only 51.9% nucleotide identity with each other and only 52.0% and 51.8% nucleotide identity, respectively, with type strain LVR 42-0/M6941, which is roughly equivalent to the nucleotide percent identity between the prototypical PRRSV and LDV strains (51.1%; Table 2). A maximum-likelihood phylogenetic tree constructed from concatenated nucleotide sequence alignments of homologous ORFs from representative arteriviruses is shown in Figure 2. The tree is consistent with established phylogenetic relationships among the arteriviruses [21], [22], supporting with high statistical confidence the close relationship between PRRSV and LDV relative to SHFV and EAV. Within the SHFV clade, SHFV-krc1 and SHFV-krc2 are highly divergent from each other and from the type strain SHFV LVR 42-0/M6941. Although SHFV-krc1 and SHFV-krc2 cluster together, statistical support for this relationship is relatively weak even with near-complete genome sequences. Overall, the degree of phylogenetic diversity within SHFV is remarkably high and is approximately equal to that between PRRSV and LDV.

**Table 2:** Pair-wise percent nucleotide identities among arteriviruses, including two new SHFV variants from Kibale red colobus (SHFV-krc1 and SHFV-krc2) and prototype *Arterivirus* strains^a^.

	SHFV-krc1	SHFV-krc2	SHFV-LVR	LDV-Plagemann	PRRSV-Lelystad	EAV-Bucyrus
SHFV-krc1	*100*					
SHFV-krc2	51.9	*100*				
SHFV-LVR	52.0	51.8	*100*			
LDV-Plagemann	44.6	42.8	43.7	*100*		
PRRSV-Lelystad	43.9	43.9	43.9	51.1	*100*	
EAV-Bucyrus	40.8	40.4	40.9	41.4	40.6	*100*

a Values are uncorrected pair-wise percent nucleotide identities of aligned, concatenated ORFs 1a, 1b, 2a, 2b, and 3–7, with reference to the EAV genome (see Figure 1). Other prototypical viruses included in the analysis are (GenBank accession numbers in parentheses): SHFV-LVR, the SHFV type strain LVR 42-0/M6941 (NC_003092.1); LDV-Plagemann strain (U15146.1); PRRSV-Lelystad strain (M96262.2); and EAV-Bucyrus strain (NC_002532.2).

## Discussion

Using direct pyrosequencing, we have demonstrated infection of a male red colobus monkey from Kibale National Park, Uganda, with two novel SHFV variants. To our knowledge, this is the first report of SHFV in a wild primate. These new viruses, SHFV-krc1 and SHFV-krc2, are highly divergent from each other and from the type strain (approximately 52% nucleotide sequence identity among the three variants). Both new viruses nevertheless contain homologs of the type strain SHFV ORFs 2a, 2b, and 3 in the same genomic positions.

Our results shed light on the natural history of SHFV. Asymptomatic infections of captive patas monkeys, vervet monkeys, and baboons support the notion that African primates are the natural hosts of SHFV [2], [3]. However, SHFV has, to our knowledge, only been isolated from these species upon their arrival in primate colonies, raising the possibility of infection during transport. The two new SHFV variants that we have discovered were isolated from a wild Ugandan red colobus monkey in a natural setting, providing conclusive evidence that wild African primates are natural hosts of SHFV.

Phylogenetic analysis of SHFV-krc1 and SHFV-krc2 demonstrate the diversity of SHFV to be higher than has been documented for any other arterivirus. Indeed, the degree of phylogenetic divergence between the two new SHFV strains and the type strain is approximately equivalent to the degree of divergence between PRRSV and LDV, which are considered different viral species. We caution, however, that phylogenetic diversity within the other arteriviruses may be underestimated due to small numbers of currently available full genome sequences. For example, divergent EAV strains have been documented [23], [24] but, at the time of this writing, full genomes of such EAV isolates were not available. Unfortunately, our attempts to align the available hypervariable partial ORF5 sequences of these divergent EAV strains with homologous regions from other arteriviruses failed due to low sequence similarity. Future studies of EAV and other as-yet undiscovered arteriviruses may demonstrate levels of within-species diversity that match or even exceed what we have documented for SHFV.

Particularly noteworthy was our finding of natural co-infection of a single primate host with two highly divergent SHFV variants. To our knowledge, no instance of co-infection of a single host with such highly divergent arteriviruses has previously been reported. Kibale red colobus may simply harbor a very diverse population of SHFV variants. However, it is also possible that SHFV-krc1 or SHFV-krc2 may have been transmitted to this animal from another species. For example, red colobus in Kibale form polyspecific associations with other primates, most frequently with red-tailed guenons (*Cercopithecus ascanius*) but also with black-and-white colobus (*Colobus guereza*), blue monkeys (*Cercopithecus mitis*), and grey-cheeked mangabeys (*Lophocebus albigena*) [25]. Because such interactions can involve close proximity and direct contact, polyspecific associations could facilitate the cross-species transmission of viruses that require close contact, such as SHFV.

Our results highlight the utility of direct pyrosequencing for detecting novel viruses and for characterizing viral genomic architecture during natural infection. The combination of random hexamer-primed reverse transcription and double-stranded cDNA synthesis used in this study obviated the need for PCR amplification to generate suitable amounts of DNA for pyrosequencing libraries. This method also prevented the potential introduction of amplification-induced error and template bias associated with whole genome amplification [26]. Despite marked divergence from each other and from type strain LVR 42-0/M694, SHFV-krc1 and SHFV-krc2 share the same 3′ genomic architecture as the type strain, consisting of additional ORFs 2a, 2b, and 3, which are not present in the other arteriviruses (Figure 1). Because our methods did not involve tissue culture, which can introduce genetic anomalies as viruses replicate in, and potentially adapt to, cells or cell lines derived from non-natural host species, we conclude that the unique 3′ genomic architecture of SHFV in comparison to the other arteriviruses is authentic and characteristic of SHFV. Future studies of isolated viruses should help clarify the stability of SHFV in cell culture and should facilitate investigations of biological differences among SHFV variants, including their ability to be detected by available serologic diagnostics tests [27].

Finally, our results have implications for primate health and conservation. The animal sampled was apparently healthy at the time of capture and, as of the time of this writing, continues to behave normally within its social group. Preliminary data (unpublished) indicate that this particular animal is also infected with the recently discovered simian immunodeficiency virus SIV-krc [20], and that other apparently healthy red colobus in the same location are infected with SHFV-krc. This evidence supports the idea that wild primates can harbor SHFV subclinically, although long-term field observations of infected and uninfected animals will be required to detect any negative effects of infection and co-infection with other viruses on host fitness. Nevertheless, our findings underscore that wild primates can be reservoirs for unknown pathogens of potential concern for global health, and that extreme care should therefore be taken when introducing primates into new environments. Indeed, we suggest that direct pyrosequencing or other similar technologies for broad viral screening might prove useful in settings such as zoos, primate colonies, sanctuaries, and primate reintroduction programs where asymptomatic carriers of novel viral pathogens might come into contact with clinically susceptible hosts.
